# Confluent Granulomas and Ulcers Lined by Epithelioid Histiocytes: New Ideal Method for Differentiation of ITB and CD? A Meta Analysis

**DOI:** 10.1371/journal.pone.0103303

**Published:** 2014-10-09

**Authors:** Juan Du, Yan-Yan Ma, Ha Xiang, You-Ming Li

**Affiliations:** 1 Department of Gastroenterology, First Affiliated Hospital of Zhejiang University School of Medicine, Hangzhou, Zhejiang Province, China; 2 Department of Pathology, First Affiliated Hospital of Zhejiang University School of Medicine, Hangzhou, Zhejiang Province, China; University Hospital Llandough, United Kingdom

## Abstract

**Background:**

There are few widely accepted criteria other than caseation, which has low sensitivity, for differentiating intestinal tuberculosis (ITB) and Crohn's disease (CD).

**Objective:**

We performed a meta-analysis to evaluate the use of confluent granulomas and ulcers lined by epithelioid histiocytes as histological methods for differentiating ITB and CD, compared with that of caseation.

**Methods:**

We searched PubMed, Medline, Embase, Web of Science, the Cochrane Library and Chinese Biomedicine Database for all relevant studies on the histological differentiation of ITB and CD. Sensitivity, specificity, and diagnostic odds ratio (DOR) were calculated for each study. Study quality and heterogeneity were assessed. Meta-regression analysis and sensitivity analyses were performed.

**Results:**

Ten randomized trials involving 316 ITB and 376 CD patients were included. The results showed that analysis of caseation showed an overall weighted area under the curve (AUC) of 0.9966, overall sensitivity and specificity were 0.21 and 1.00, respectively, with a positive likelihood ratio (+LR) of 10.79, negative likelihood ratio(-LR) of 0.82 and DOR of 13.74. Confluent granulomas had a lower overall weighted AUC of 0.9381, sensitivity and specificity were 0.38 and 0.99, respectively, with a +LR of 16.29, -LR of 0.65 and DOR of 26.52. Overall weighted AUC for ulcers lined by epithelioid histiocytes was 0.9017, sensitivity and specificity were 0.41 and 0.94, respectively, with a +LR of 6.46, -LR of 0.54 and DOR of 13.17. Significant heterogeneity was noted for the studies. Meta-regression analysis showed that study source, publication year, size, design and quality did not affect heterogeneity.

**Conclusion:**

Confluent granulomas and ulcers lined by epithelioid histiocytes are helpful in distinguishing ITB from CD, which may provide a new method, other than caseating granulomas and acid-fast bacilli, to differentiate ITB and CD in mucosal biopsies.

## Introduction

Crohn's disease (CD) is a multifactorial inflammatory bowel disease (IBD), which results in idiopathic chronic inflammation in the gastrointestinal (GI) tract. The pathogenesis of CD is incompletely understood. CD is traditionally characterized according to clinical, radiological, endoscopic and histological features. However, in clinical practice, the observed cobblestone appearance on endoscopy was calculated at a sensitivity between 27.7 to 34.1% when diagnosing CD [1]–[2]. At present, the incidence of CD is higher year by year in developing countries, especially in China and India [3].

Intestinal tuberculosis (ITB) is caused by *Mycobacterium tuberculosis*, and is often seen secondary to pulmonary tuberculosis. Although ITB is rare in developed countries, it is prevalent in developing countries [4]. ITB and CD are both chronic granulomatous disorders with similar clinical presentation, morphology and pathology. They share many common immune pathways of pathogenesis, which includes, triggering potent adaptive TH1 cytokine responses resulting in granuloma formation [5]. The differentiation between ITB and CD is very important, as the treatment of each diseas is different.

A comprehensive analysis of clinical, endoscopic and histological features may contribute to an accurate diagnosis Of the diagnostic techniques available, endoscopy combined with biopsy are the best choice for the physician [6]. We carried out a meta-analysis to assess the diagnostic characteristics of confluent granulomas and ulcers lined by epithelioid histiocytes during screening to histologically differentiate ITB from CD.

## Materials and Methods

### Search Strategy

We searched Pubmed, Medline, Google Scholar, Embase, Web of Science, Chinese Biomedicine Database, and the China Journal Full Text Database for data published from January 1995 to June 2013. We restricted our search to studies published in English and Chinese only. The search terms included: (“Crohn's disease or Crohn or CD” AND “intestinal tuberculosis or ITB” AND “biopsy or biopsies or histology or histological or pathology or pathological or histopathology or histopathological”) and (“Crohn Disease” [Mesh] AND “Tuberculosis” [Mesh]) and (biopsy or biopsies or histology or histological or pathology or pathological or histopathology or histopathological). We also searched the reference lists of each selected study manually.

### Inclusion and Exclusion Criteria

Inclusion criteria were as follows: 1. Chinese or English studies; 2. Studies comparing the histopathology of ITB and CD; 3. Prospective or retrospective studies; 4. The diagnostic criteria for CD: In the English studies diagnosis was according to the European Crohn's and Colitis Organisation (ECCO) [7]. In the Chinese studies, diagnosis was according to the suggested guidelines for the diagnosis and treatment of inflammatory bowel disease (IBD), which were approved in China in 2001 [8] or the Consensus on the management of inflammatory bowel disease in China in 2007 [9]. The diagnostic criteria for ITB: a. Caseous necrosis on histology (including intestine, peritoneum and lymph nodes); b. Culture positivity; c. radiologic, colonoscopic, and/or operative evidence of ITB with proven tuberculosis elsewhere. d. TB PCR positivity in serum. e. Response to anti-tuberculosis therapy. The following characteristics were extracted from each selected study: 1. Abstract, review, commentary and case report; 2. Duplication of data published; 3. Incomplete data; 4. Did not meet one of the inclusion criteria.

### Assessment of Methodological Quality

Two reviewers independently coded each study for the presence or absence of the methodologic limitations described above. Disagreement was resolved by discussion. Basic data as well as a number of quality indicators were systematically registered. They included first author, year of study, country, setting (inpatients or outpatients), sample size, design and primary data.

Study quality was assessed by two reviewers using the original QUADAS (Quality Assessment of studies of Diagnostic Accuracy included in Systematic reviews) checklist. Each item was scored as “yes” “no” or “unclear”, in which a score of 1 was given when a criterion was fulfilled, 0 if a criterion was unclear, and −1 if the criterion was not achieved. Disagreement was resolved by discussion.

### Analysis

Data were used as reported in the original article. Data were extracted by one reviewer, and checked by a second. Calculations were carried out using a four-fold table (table 1). Some of these calculations were based on unpublished data provided by the author at our request. We calculated sensitivity (Sens), specificity (Spec), positive (PPV), and negative (NPV) predictive value, likelihood ratios (LR) and the area under the ROC curve (AUC) using Meta-Disc 1.4 software.

**Table 1:** Four-fold table.

		Ill	Illness	Total
Diagnostic test	Positive	a	b	a+b
	Negative	c	d	c+d
	Total	a+c	b+d	a+b+c+d

### Calculation Formula



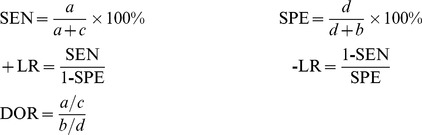



We used Cochran's Q heterogeneity statistic and the quantity, I2, to determine the percentage of total variation across the studies due to heterogeneity rather than to chance. When P≤0.1 or I2 was more than 50%, we explored possible reasons for heterogeneity.

Statistical methods used in the meta-analysis included the fixed effects model (Mantel-Haenszel, Peto and Genera1 Variance-Based) and the random effects model (Dersimonian and Laird). We used the Mantel-Haenszel fixed effects model when there was no heterogeneity, otherwise the Dersimonian and Laird random effects model was used. Meta-regression, sensitivity and sub-group analyses were also performed.

As publication bias is an important concern in meta-analyses of diagnostic studies [10], we used funnel plots and the Egger test [11] to assess potential publication bias.

## Results

**Figure 1:**
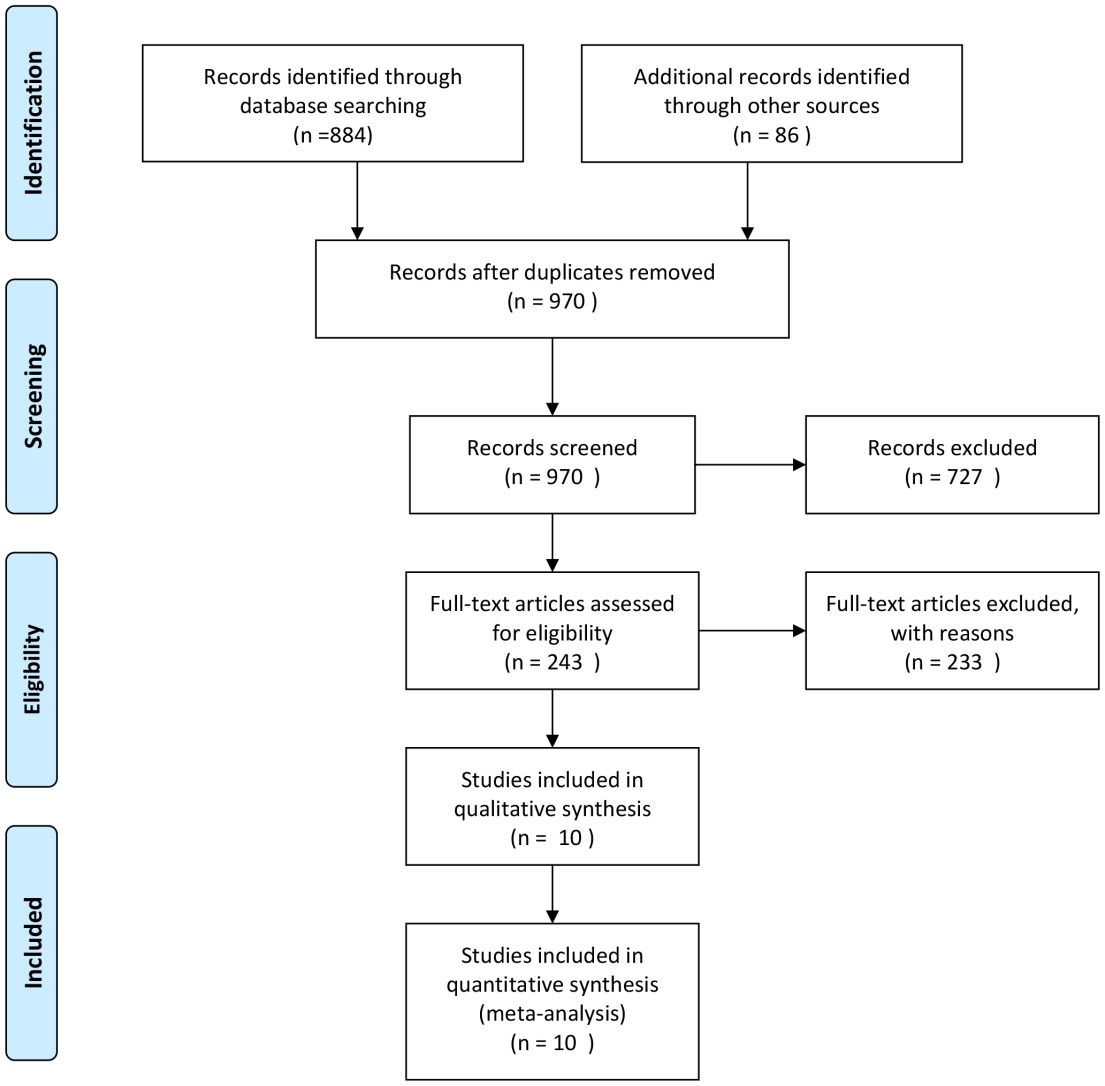
Selection of studies.

The study included the results of electronic searches up to June 2013. A total of 970 papers were identified. Of these, 727 were excluded after reading the titles and abstracts. The final analysis included eleven studies. The remaining studies were excluded due to repeated publication, did not meet the inclusion criterion or had insufficient data (Figure 1).

We included 10 studies involving 692 patients, of which 316 had ITB and 376 had CD. The characteristics, designs and QUADAS scores are presented in Table 2.

**Table 2:** Characteristics of the included studies.

Study	Country & year	Sample size	Study type	Blinding	Follow-up	QUADAS
Miao YL [12]	China 2002	60(30/30)	retrospective	no	yes	9
Zhou ZY [13]	China 2006	60(30/30)	retrospective	no	yes	9
Gu Q [14]	China 2009	67(34/33)	retrospective	no	yes	9
Li Y [15]	China 2011	84(19/65)	prospective	no	yes	13
Dutta AK [16]	India 2011	60(30/30)	prospective	no	yes	11
Amarapurkar DN [17]	India 2008	52(26/26)	prospective	no	yes	12
Makharia CK [18]	India 2009	106(53/53)	retrospective	no	yes	11
Yu HS [19]	China 2012	96(43/53)	retrospective	no	yes	11
Pulimood [20]	India 2005	64(33/31)	retrospective	no	yes	9
Kirsch [21]	Canada 2006	43(18/25)	retrospective	no	yes	10

In total, nine studies mentioned the differential diagnosis of ITB and CD using caseation. Meta-regression showed that the study source, publication year, size, design and quality did not affect heterogeneity. Sensitivity analysis showed that the DOR decreased when the studies by Zhou ZY and Miao YL were removed. These two excluded studies may affect our final results. Funnel plots and the Egger [Pr>|z| = 0.133 and P>|t| = 0.712] test both showed that there was no publication bias.

**Figure 2:**
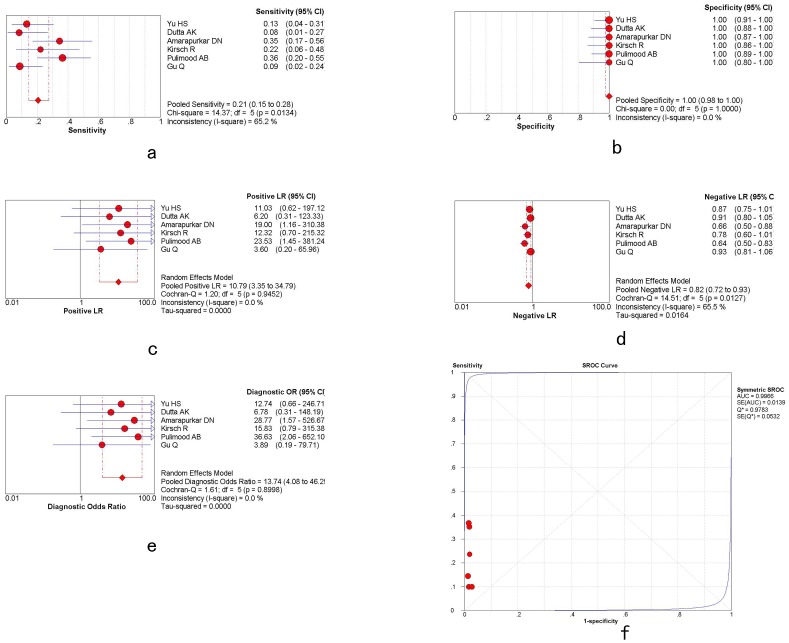
The plots of caseation. a,g,m: overall weighted sensitivity; b,h,n: overall weighted specificity; c,o: overall weighted positive likelihood ratios; d,j,p: overall weighted negative likelihood ratios: e,k,q: overall weighted diagnostic OR; f,l,r: overall weighted AUC. (red spots represent the value of sensitivity, specificity, positive likelihood ratios, negative likelihood ratios, diagnostic OR and AUC. Length of single lines represents the confidence interval. Diamonds represent the combined effects of the amount estimated.)

Overall weighted AUC for caseation in distinguishing ITB and CD was 0.9966 (SE 0.0139), Q* = 0.9783, with significant heterogeneity between the studies (Q-value 1.61, P = 0.8998). The sensitivity and specificity were 0.21 [95%CI: 0.15–0.28] and 1.00 [95%CI: 0.98–1.00], respectively, with a +LR of 10.79 [95%CI: 3.35–3.79], -LR of 0.82 [95%CI: 0.72–0.93] and diagnostic OR (DOR) of 13.74 [95%CI: 4.08–46.25], shown in figure 2a–f and table 3.

**Table 3:** Overall weighted sensitivity, specificity, positive likelihood ratios, negative likelihood ratios, diagnostic OR and AUC of caseation, confluent granulomas and ulcers lined by epithelioid histiocytes.

	Sensitivity	Specificity	+LR	-LR	DOR	AUC
Caseation	0.21 (0.15–0.28)	1.00 (0.98–1.00)	10.79 (3.35–34.79)	0.82 (0.72–0.93)	13.74 (4.08–46.25)	0.9966
Confluent granulomas	0.38 (0.30–0.47)	0.99 (0.95–1.00)	16.29 (5.66–46.87)	0.65 (0.57–0.75)	26.52 (8.66–81.23)	0.9381
Ulcers lined by epithelioid histiocytes	0.41 (0.32–0.51)	0.94 (0.88–0.98)	6.46 (1.80–23.17)	0.54 (0.28–1.04)	13.17 (2.21–78.60)	0.9017

Five studies mentioned the differential diagnosis of ITB and CD using confluent granulomas. Meta-regression analysis did not identify heterogeneity. Sensitivity analysis showed that the DOR did not change when each of the studies were removed. Funnel plots and the Egger test [Pr>|z| = 0.086; P>|t| = 0.060] both showed that there was no publication bias.

**Figure 3:**
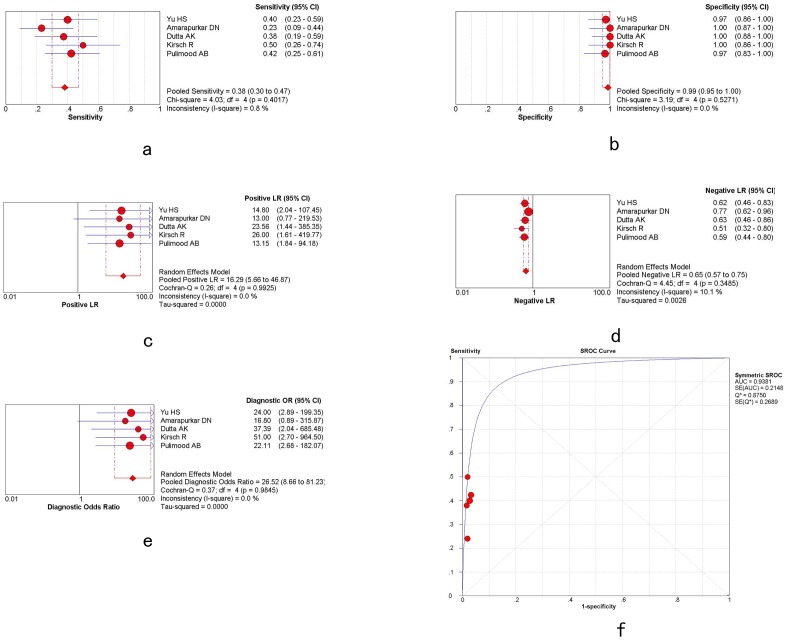
The plots of confluent granulomas.

Overall weighted AUC for confluent granulomas in distinguishing ITB and CD was 0.9381 (SE 0.2148), Q* = 0.8750, with significant heterogeneity between the studies (Cochran-Q = 0.37; P = 0.9845>0.05). The sensitivity and specificity were 0.38 [95%CI: 0.30–0.47] and 0.99 [95%CI: 0.95–1.00], respectively, with a +LR of 16.29 [95%CI: 5.66–46.87], -LR of 0.65 [95%CI: 0.57–0.75] and DOR of 26.52 [95%CI: 8.66–81.23], shown in figure 3a–f and table 3.

Only three studies mentioned the differential diagnosis of TB and CD using ulcers lined by epithelioid histiocytes. Meta-regression showed that the study source, publication year, size, design and quality did not affect heterogeneity. Sensitivity analysis showed that the DOR did change when the study by Makharia was removed. Funnel plots and the Egger test [Pr>|z| = 1.000; P>|t| = 0.419] both showed that there was no publication bias.

**Figure 4:**
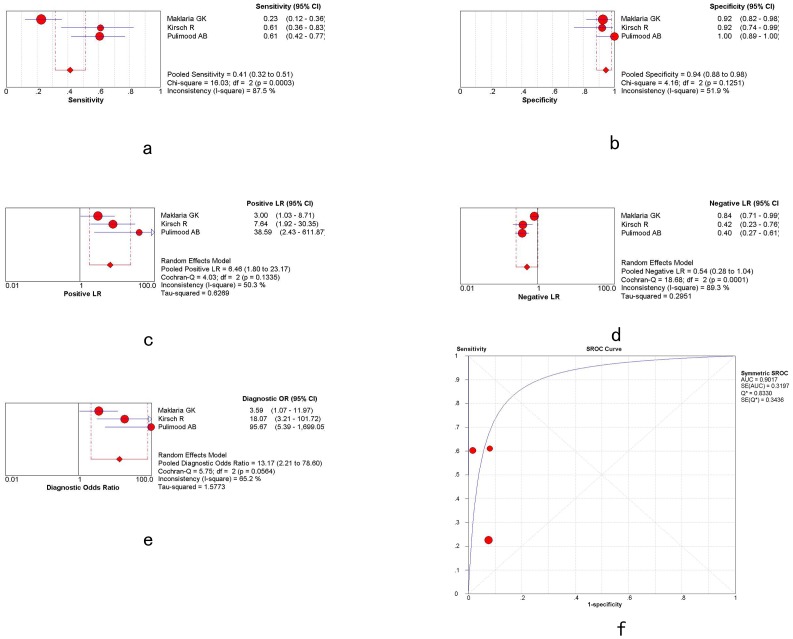
The plots of ulcers lined by epithelioid histiocytes.

Overall weighted AUC for ulcers lined by epithelioid histiocytes in distinguishing ITB and CD was 0.9017 (SE 0.3197), Q* = 0.8330, with significant heterogeneity between the studies (Cochran-Q = 5.75; P = 0.0564>0.05). The sensitivity and specificity were 0.41 [95%CI: 0.32–0.51] and 0.94 [95%CI: 0.88–0.98], respectively, with a +LR of 6.46 [95%CI: 1.80–23.17], -LR of 0.54 [95%CI: 0.28–1.04] and DOR of 13.17 [95%CI: 2.21–78.60], shown in figure 4a-f and table 3.

## Discussion

The pathogenesis of CD is incompletely understood and is potentially caused by interactions between genetic and environmental factors. The prevalence of CD appears to be higher in Northern and Western Europe as well as North America, but lower in Africa, South America and Asia [22]. The highest annual incidence of CD was 12.7 per 100,000 person-years in Europe, 5.0 person-years in Asia and the Middle East, and 20.2 per 100,000 person-years in North America [23]. However, over the past few decades, the geographical incidence of CD has varied considerably worldwide, especially in Asia, including China and India [24]–[25], causing heated debate and discussion among researchers around the world. ITB and CD have similar clinical, endoscopic and histological features, which makes it difficult to distinguish between them, especially in regions where the incidence of both diseases are relatively high, such as China and India.

Tuberculosis is diagnosed by acid-fast staining, culture positivity and the presence of caseous granulomas, which are rarely found in ITB [26]–[27]. Thus, the rate of misdiagnosis between ITB and CD is high [28]–[29]. A study in India [30] investigated 100 cases with resected intestinal specimens, including 22 request forms with clinical suspicion of ileocecal tuberculosis. The results showed that only 6 cases of ITB were verified. ITB can mimic various diseases both clinically and sometimes morphologically, especially CD. Another study from Korea [31] reported that approximately 50% of CD patients received anti-tuberculosis treatment before confirmation of CD.

The differentiation between ITB and CD is very important as the treatment of each disease is different. If wrongly treated, there are risks of drug toxicity and additional complications. In particular, if corticosteroids are used to treat ITB, the focus of infection may spread, or even result in death. Therefore, it is important to identify a new method, other than caseating granulomas and acid-fast bacilli, to differentiate ITB and CD in mucosal biopsies.

**Figure 5:**
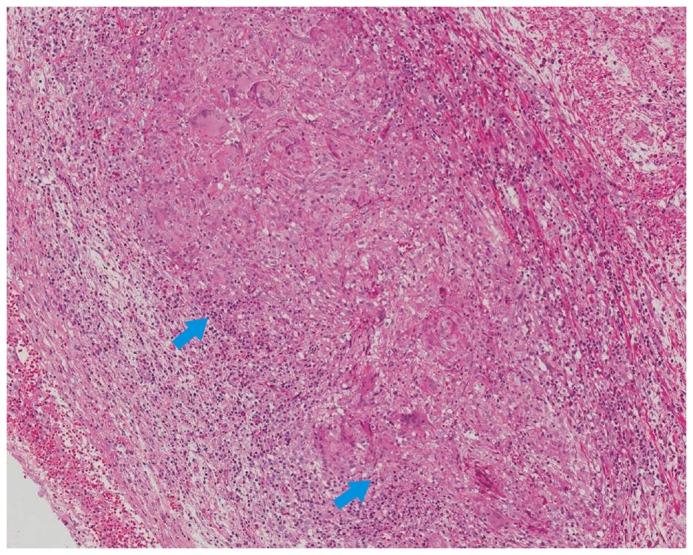
Appendectomy specimens showing confluent garnuloma.HE.X200.

**Figure 6:**
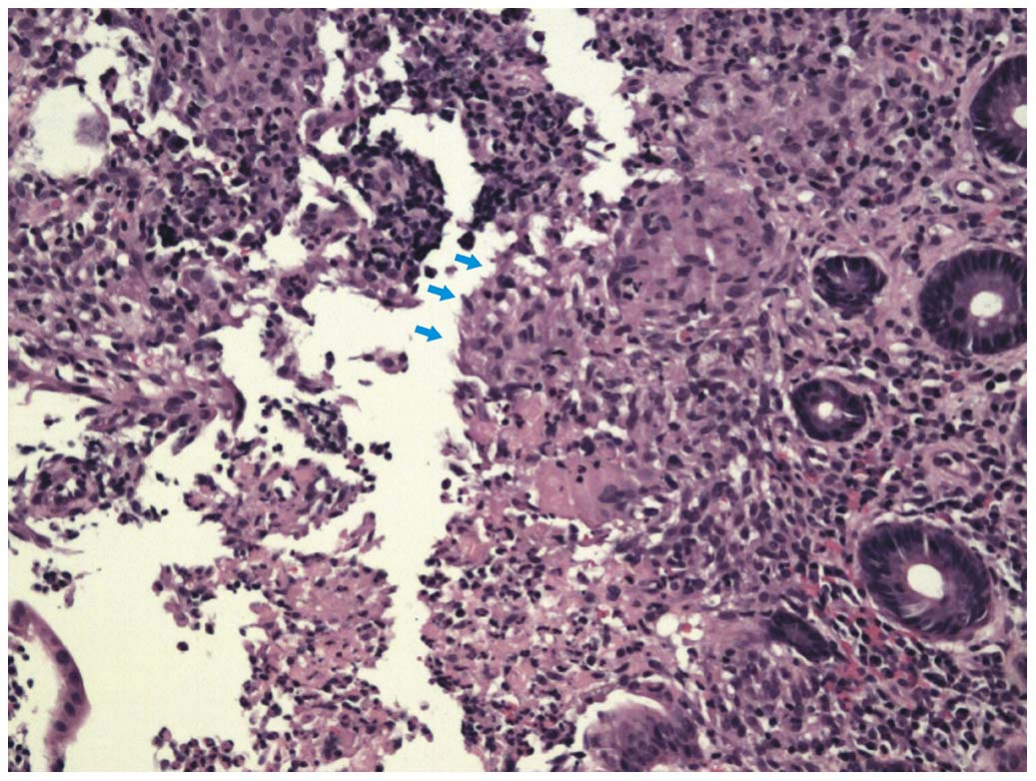
Endoscopic biopsy specimens showing ulcers lined by epithelioid histiocytes (arrows).HE.X200.

In this study, we found that confluent granulomas and ulcers lined by epithelioid histiocytes were helpful in distinguishing ITB from CD. Confluent granulomas are the merging of the boundaries of adjacent granulomas [20] (shown in figure 5) and ulcers lined by epithelioid histiocytes are large deep ulcers with granulation tissue in the ulcer base with a band of epithelioid histiocytes at the periphery [18] (shown in figure 6). We compared confluent granulomas and ulcers lined by epithelioid histiocytes with caseation, which is the ideal method, to explore the value of confluent granulomas and ulcers lined by epithelioid histiocytes in the diagnosis of ITB.

Pathologic features such as location and number of granulomas, caseation necrosis, microgranulomas, confluent granulomas, ulcers with bands of epithelioid histiocytes, granulomas with lymphoid cuffs and disproportionate submucosal inflammation are known to be important in differentiating ITB and CD. The results of our study showed that compared with caseation necrosis, the sensitivity of confluent granulomas and ulcers lined by epithelioid histiocytes was 0.38, 0.41 vs 0.21, and the specificity was 0.99, 0.94 vs 1.00, respectively. A perfect predictor should have 100% sensitivity and 100% specificity, however, this is rarely observed. From our data, it can be seen that the sensitivity of confluent granulomas and ulcers lined by epithelioid histiocytes was almost twice that of caseation, while the specificity was similar.

Further analysis showed that the AUC of confluent granulomas and ulcers lined by epithelioid histiocytes was 0.9381 and 0.9017, respectively. The AUC of caseation necrosis was 0.9966, which was much higher than confluent granulomas and ulcers lined by epithelioid histiocytes. The AUC is commonly used to assess diagnostic value. If the AUC value is more than 0.9, then the diagnostic accuracy is very high.

A limitation of our study is that microgranulomas, granulomas >5 per section, granulomas in submucosa, disproportionate submucosal inflammation, granulation tissue, size of granulomas and inflammation beyond the site of macroscopically evident lesions were not considered in this meta analysis, as the article space is limited. Our meta analysis only included the studies which were published. We may have lost information, due to unpublished results, resulting in an analytical bias. Additionally, expert opinions vary by region. This provides an additional bias to our study, due to the current lack of universal standard when performing endoscopic diagnosis and histological analysis.

## Conclusion

This meta-analysis showed that confluent granulomas and ulcers lined by epithelioid histiocytes have high diagnostic precision in differentiating ITB from CD. More studies are needed to assess the best method in clinical practice.
